# Deacylation of Calcium‐Dependent Antibiotics from *Streptomyces violaceoruber* in Co‐culture with *Streptomyces* sp. MG7‐G1

**DOI:** 10.1002/cbic.202000404

**Published:** 2020-07-20

**Authors:** Kathrin Schindl, Deepika Sharma, Dieter Spiteller

**Affiliations:** ^1^ Chemical Ecology/Chemical Biology University of Konstanz Universitätstrasse 10 78457 Konstanz Germany

**Keywords:** antibiotics, lipopeptides, mass spectrometry, microbe–microbe interactions, natural products

## Abstract

When *Streptomyces violaceoruber* grows together with *Streptomyces* sp. MG7‐G1, it reacts with strongly induced droplet production on its aerial mycelium. Initially the metabolite profile of droplets from *S. violaceoruber* in co‐culture with *Streptomyces* sp. MG7‐G1 was compared to samples from *S. violaceoruber* in single‐culture by using high‐performance liquid chromatography‐mass spectrometry (HPLC‐MS). Then, the exudate from agar plates of co‐cultures and single cultures (after freezing and thawing) was also analysed. Several compounds were only observed when *S. violaceoruber* was grown in co‐culture. Based on their high‐resolution ESI mass spectra and their comparable retention times to the calcium‐dependent antibiotics (CDAs) produced by *S. violaceoruber*, the new compounds were suspected to be deacylated calcium‐dependent antibiotics (daCDAs), lacking the 2,3‐epoxyhexanoyl residue of CDAs. This was verified by detailed analysis of the MS/MS spectra of the daCDAs in comparison to the CDAs. The major CDA compounds present in calcium ion‐supplemented agar medium of co‐cultures were daCDAs, thus suggesting that *Streptomyces* sp. MG7‐G1 expresses a deacylase that degrades CDAs.

## Graphical abstract

**Influencing the neighbour**: In co‐culture *Streptomyces* sp. MG7‐G1 induces *Streptomyces violaceoruber* to produce droplets on its areal mycelium. Moreover, the calcium‐dependent antibiotics from *S. violaceoruber* are deacylated, in particular when the concentration of Ca^2+^ ions is high.

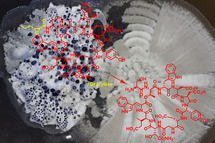

## Introduction

In nature, almost all organisms live together with others and often strongly influence each other. For example, neighbours may compete against each other for resources and organisms may share nutrients or protect their partners. Microorganisms use chemical compounds to mediate their interactions and ensure their survival.^[1]^

For decades the highly diverse secondary metabolites from microorganisms, in particular from actinomycetes, have been studied because of their invaluable potential for applications in medicine.^[2]^ However, the biological functions of such secondary metabolites in their ecological context have only recently gained interest.^[1]^ Routine genome sequencing has revealed that many microorganisms produce many more secondary metabolites than can be isolated from them when they are cultivated in single cultures under optimised growth conditions in the lab,^[3]^ in part because many compounds are clearly only needed in specific situations. Consequently, varying growth conditions^[4]^ or mimicking natural conditions, for example, by co‐cultivation with other organisms,[^1^, ^5^] can help to reveal thus far unknown metabolic functions of microbial genes, and contribute to an in‐depth understanding of the chemical ecology of microorganisms.

Apart from improving the basic understanding of microorganisms, revealing the hidden metabolic potential of microbes can lead to the discovery of new strategies to address severe emerging threats, such as agricultural pests,^[6]^ pollution^[7]^ and life threatening infections.^[8]^ Due to the evolution of multidrug‐resistant clinical strains, such as methicillin‐ and vancomycin‐resistant staphylococci, enterococci or streptococci,^[9]^ we now face a drastic increase in fatalities caused by infectious diseases that are expected to further rise dramatically in the coming decades.^[8]^

Scientists have identified multiple novel secondary metabolites by co‐cultivation experiments.[^1^, ^5^] In order to reveal unknown metabolic functions, we started to grow *Streptomyces* strains together with other microorganisms and screened them for morphological and chemical changes. We co‐cultivated the strains by alternately spotting them on agar plates. Our screening led to the observation that *Streptomyces* sp. MG7‐G1 provoked *Streptomyces violaceoruber* to form blue droplets on its aerial mycelium in large numbers.^[10]^ The formation of droplets by some streptomycetes is well known. Colonies with droplets are often depicted because of their aesthetic appearance, and droplet production was even used as a criterion for species description.^[11]^ Usually, the droplets are produced infrequently.^[10]^ However, when bacteria such as *Streptomyces* sp. MG7‐G1 and *S. violaceoruber* grow together, this leads to strongly induced and reliable droplet formation. *Streptomyces* sp. MG7‐G1 releases a high amount of ammonia that triggers droplet formation in *S. violaceoruber*.^[10]^ Because ammonia from *Streptomyces* sp. MG7‐G1 provokes *S. violaceoruber* to form more than 15 times more droplets than when grown alone, we investigated whether the interaction of both strains resulted in an alteration of their metabolite profiles and led to the production of thus far unknown secondary metabolites. Both strains appeared to grow perfectly fine together although ammonia at the concentration released by some *Streptomyces* strains can also exert antimicrobial activity.^[12]^ Here, we report that co‐cultivation of *S. violaceoruber* with *Streptomyces* sp. MG7‐G1 results in the deacylation of calcium‐dependent antibiotics (CDAs)^[13]^ from *S. violaceoruber*.

## Results and Discussion

**Figure 1:**
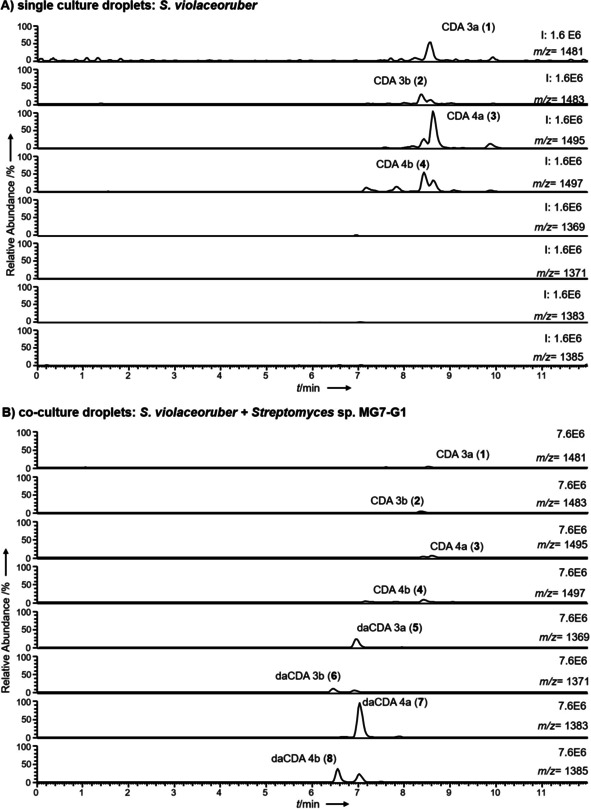
Comparison of ion trace RP18 UHPL‐chromatograms of droplets from A) *S. violaceoruber* grown in single‐culture and B) *S. violaceoruber* grown together with *Streptomyces* sp. MG7‐G1 on SFM agar plates for 15 days. All ion traces are depicted at the same intensity scale for A) 1.6×10^6^ and B) 7.6×10^6^, respectively.

In order to study the interactions between *Streptomyces* sp. MG7‐G1 and *S. violaceoruber*, we compared the metabolite profiles of *S. violaceoruber* grown in co‐culture with *Streptomyces* sp. MG7‐G1 to samples from *S. violaceoruber* grown alone (Figure S1 in the Supporting Information). Comparing the LC‐MS profiles of droplets from single cultures with those of the co‐cultures, we detected several new peaks in the co‐cultures (Figure 1).

**Scheme 1:**
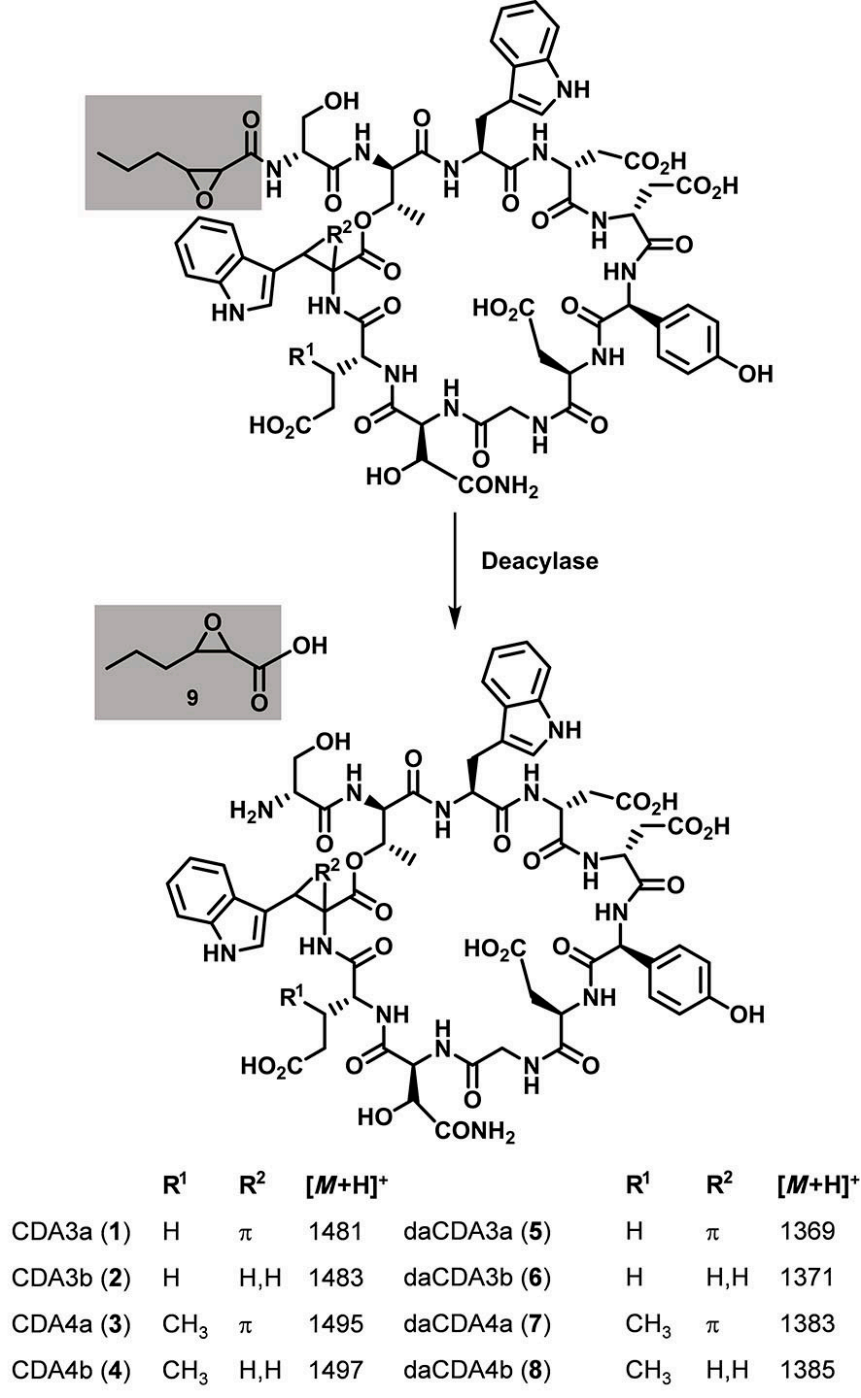
Conversion of CDAs to deacylated CDAs when *S. violaceoruber* grows together with *Streptomyces* sp. MG7‐G1. The 2,3‐epoxyhexanoyl residue is shaded in grey.

In addition to the calcium‐dependent antibiotics (CDAs) CDA3a (**1**, *m/z* 1481), CDA3b (**2**, *m/z* 1483), CDA4a (**3**, *m/z* 1495), and CDA4b (**4**, *m/z* 1497), all of which are well known from *Streptomyces coelicolor* A3(2)^[13a]^ and also produced by the closely related *S. violaceoruber* DSM 40783,^[14]^ several new compounds **5**, **6**, **7** and **8** were found in samples from the co‐cultures that eluted at a similar retention time as the CDAs (Scheme 1, Figure 1). CDAs are nonribosomal lipopeptides that slightly differ in their peptide core, but all contain a 2,3‐epoxyhexanoyl moiety as their lipid residue.^[13a]^ Strikingly, the molecular mass of compounds **5**, **6**, **7** and **8** differed from the CDAs **1**, **2**, **3** and **4**, respectively, by 112 amu, which is consistent with the loss of the 2,3‐epoxyhexanoyl moiety of CDAs in each case.

**Figure 2:**
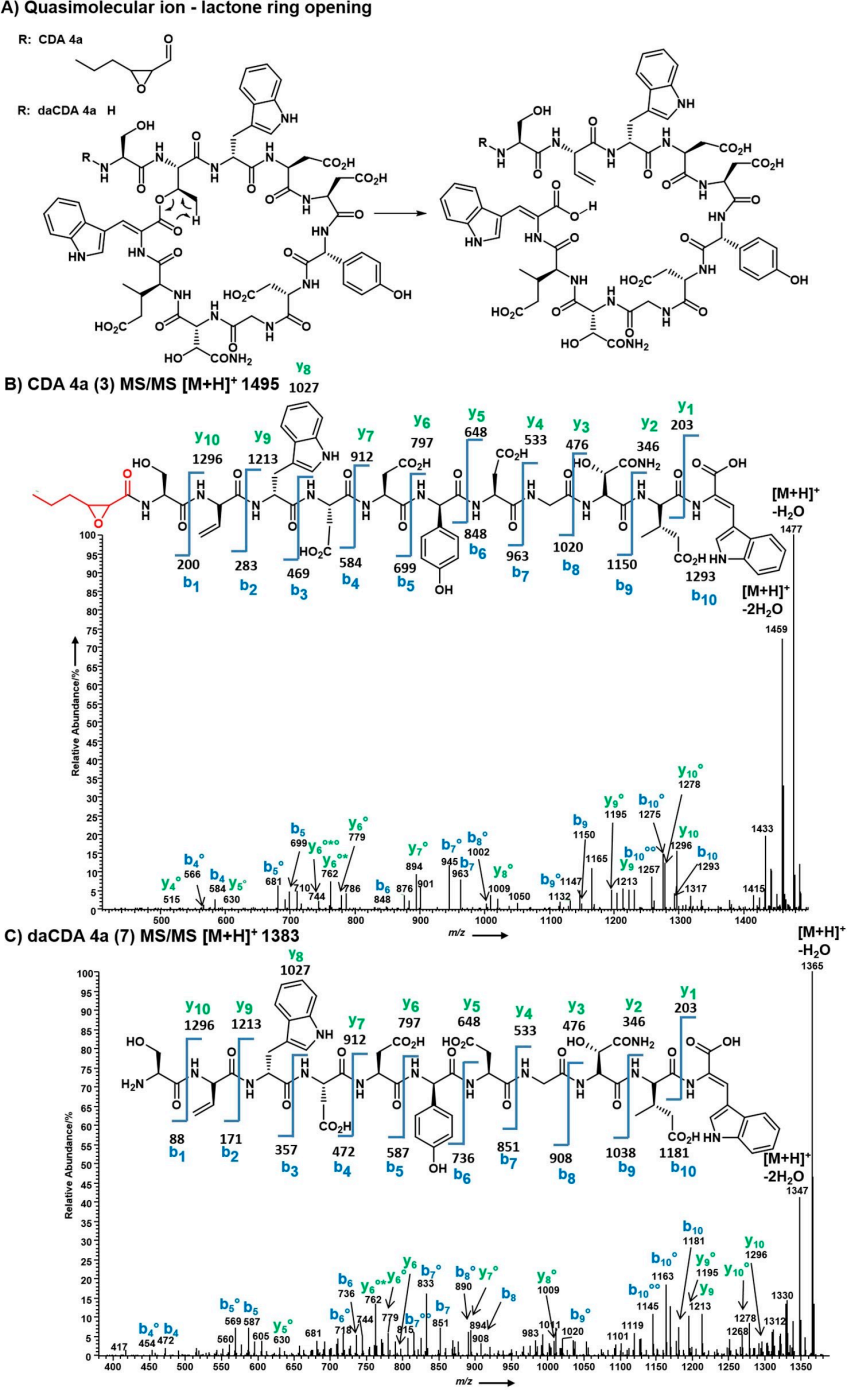
A) Ring opening of the quasimolecular ions [*M*+H]^+^ of CDAs and daCDAs followed by the fragmentation of the linear quasimolecular ions of CDA4a (**3**) and daCDA4a (**7**). ESI‐MS/MS spectra of B) CDA4a (**3**) and C) daCDA4a (**7**). The loss of water is indicated by superscripted circles, the loss of ammonia by asterisks. The b‐ion‐derived fragments of **7** exhibit a mass difference of 112 u compared to **3**; this corresponds to the missing 2,3‐epoxyhexanoyl residue (R).

High‐resolution electrospray ionisation mass spectrometry (HRMS‐ESI) in combination with the isotope pattern analysis confirmed the molecular formulas of CDAs and deacylated CDA (daCDAs, see the Supporting Information). In order to further verify the deacylation of CDAs we analysed the MS/MS fragmentation pattern of daCDAs in comparison to the CDAs using high‐resolution electrospray tandem fragmentation mass spectrometry of the [*M*+H]^+^ quasimolecular ions. Hojati et al. studied the MS/MS fragmentation of CDAs previously.^[13a]^ Both CDAs and daCDAs quasimolecular ions first undergo an opening of their lactone ring,^[13a]^ resulting in linear [*M*+H]^+^ peptide ions (e. g., CDA4a (**3**) *m/z* 1495 and daCDA4a (**7**) 1383, Figure 2A). These ions fragment into the well‐established b‐ and y‐ions of peptides, that allow following the amino acid sequence of the peptides.^[15]^

For our MS/MS experiments we first focused on the most abundant CDAs and the suspected daCDAs of *S. violaceoruber* in co‐culture with *Streptomyces* sp. MG7‐G1: CDA4a (**3**; Figure 2B) and daCDA4a (**7**; Figure 2C).

The MS/MS peptide fragmentation patterns of CDA4a (**3**) and daCDA4a (**7**) immediately confirmed that those compounds were closely related to each other. Both compounds exhibited similar fragmentation patterns with a series of common fragments (Figure 2). Due to consecutive losses of water and sometimes ammonia from the b and y ions the MS/MS fragmentation is more complex than that of simple linear peptides. The mass difference of 112 amu between CDA4a (**3**) and daCDA4a (**7**) – corresponding to the 2,3‐epoxyhexanoyl side chain – was observed for the b‐ion fragments b4, b5, b7, b8, b9, and b10, proving the loss of the 2,3‐expoxyhexanoyl residue in daCDA4a (**7**). The y ions y6, y7, y9, and y10 complemented our deductions (Figure 2B,C). Because of the “aspartic acid effect”,^[15]^ which denotes the preferential fragmentation of the peptide backbone at polar amino acid moieties, the b‐ions b5, b7, b10 were preferably generated. Similar MS/MS patterns were observed for the related CDAs and daCDAs (Supporting Information).

Because CDAs were previously obtained from the exudate after freezing and thawing of spent medium agar plates,^[13b]^ we also collected the exudates from *S. violaceoruber* single cultures as well as *S. violaceoruber*/*Streptomyces* sp. MG7‐G1 co‐cultures and analysed them by LC‐MS. As expected in the exudate of the co‐cultures the daCDAs were found as dominant CDA compounds (Figure S10). Indeed compounds from the agar plates can be transported into the droplets as our experiments with fluorescein in the agar medium clearly demonstrate (Figure S11). Thus, the CDAs/daCDAs ratio in the droplets somewhat reflects that in the agar medium. In the exudate samples of *Streptomyces* sp. MG7‐G1 no CDAs and no daCDAs were found (Figure S12). CDAs and daCDAs were present in co‐culture droplets and exudate in various amounts. Generally, the ratio of daCDAs/CDAs increased over 20 days of co‐cultivation.

In order to exert their antibiotic activity, CDAs require Ca^2+^ ions.^[13b]^ Because the observed deacylation of CDAs likely comprises a resistance mechanism so that *Streptomyces* sp. MG7‐G1 can grow together with *S. violaceoruber*, we investigated how varying Ca^2+^ ion concentrations in the SFM medium affected deacylase activity in co‐cultures of *S. violaceoruber* and *Streptomyces* sp. MG7‐G1. After 20 days of growth in co‐culture supplemented with 1 mM Ca^2+^ ions the peak area ratio of daCDAs/CDAs was approximately 10 : 1. However, at high Ca^2+^ ion concentrations (10 or 20 mM) often few to no CDAs and sometimes even no daCDAs could be detected; this is caused due to additional effects Ca^2+^ ions exert on the physiology of *Streptomyces* strains. Strongly reduced production of secondary metabolites in presence of high amounts of Ca^2+^ ions has been observed previously in *S. coelicolor*.^[25]^

Lipopeptides from bacteria and fungi bear nonpolar acyl moieties, which are attached to the polar peptide. They are produced by nonribosomal peptide biosynthesis and comprise a class of natural products that is of high pharmacological interest.^[16]^ Lipopeptides such as daptomycin,^[17]^ echinocandins,^[18]^ friulimicin B,^[19]^ amphomycins,^[20]^ laspartomycins,^[21]^ the A54145 complex,^[22]^ malacidins^[23]^ and the CDAs^[13b]^ exhibit potent antimicrobial activity. Daptomycin^[17a]^ is currently used as an antibiotic of last resort to treat severe Gram‐positive infections. The acyl residue of lipopeptides is crucial for their interaction with the lipid membranes of target organisms.[^17b^, ^24^]

Thus, the observed deacylation of CDAs in co‐cultures of *Streptomyces* sp. MG7‐G1 and *S. violaceoruber* most likely serves *Streptomyces* sp. MG7‐G1 to inactivate CDAs and coexist well with *S. violaceoruber* as indicated by its equal growth performance in co‐culture and in single culture. In line with this, we observed strong deacylase activity after supplementation of Ca^2+^ ions. The ratio of daCDA/CDA in the agar medium was always higher than their ratio in the droplets also suggesting that deacylase activity is needed for *Streptomyces* sp. MG7‐G1 to inactivate CDAs in the area where it comes close to *S. violaceoruber*.

However, there are also some examples of natural products, such as telomycins^[26]^ and pyoverdin I,^[27]^ in which precursors with acyl side chains are formed, that are then modified by the producing organism. Thus the possibility that *S. vialoceoruber* deacylates CDAs – triggered by *Streptomyces* sp. MG7‐G1 – cannot be excluded at the moment although it is much less likely than the deacylation of CDAs by *Streptomyces* sp. MG7‐G1. Only a deacylase knock‐out mutant may finally prove the suspected role of deacylation of CDAs as a resistance mechanism of *Streptomyces* sp. MG7‐G1.

So far, only few lipopeptide deacylases have been studied.[^24^, ^28^] The lipopeptide deacylase from *Actinoplanes utahiensis* has been used to remove the native acyl residues of several lipodepsipeptides.[^17b^, ^29^] After removal of the acyl residue, lipodepsipeptides with different acyl side chains were synthesised in order to vary their pharmacological properties. Daptomycin, which acts against multi drug resistant pathogens, such as methicillin‐resistant *Staphylococcus aureus* and vancomycin‐resistant enterococci, was developed using this approach.[^16^, ^17^] Similarly, acyl‐chain‐optimised derivatives of other lipopeptides, such as echinocandins,^[29a]^ the A54145 complex,^[29b]^ and laspartomycins,^[29c]^ have been produced and are either already used in the clinic or are currently in preclinical development.^[16]^

Because the choice of lipopeptide deacylases is limited, it was interesting to observe that CDAs were deacylated in co‐cultures of *S. violaceoruber* and *Streptomyces* sp. MG7‐G1. The CDA deacylase removes the 2,3‐epoxydecanoyl moiety from the peptide core of CDAs and might thus be useful to optimise their antibiotic activity by targeted acyl chain modifications. CDAs are quite unique among the cyclic lipopeptides because they only occur with the epoxyhexanoyl residue.^[30]^ In contrast, other lipopeptides have several different acyl moieties. This specificity is the reason why the acyl chain of CDAs has thus far been difficult to modify. In feeding experiments, in which the nonribosomal peptide biosynthesis gene cluster was modified, products with very limited acyl chain variability were obtained. Thus, modified CDAs containing acetyl,^[31]^ butanoyl,^[32]^ pentanoyl,^[30]^ and hexanoyl^[30]^ acyl residues were produced. A CDA deacylase instead would allow the selective deacylation of CDAs and render them amenable to a broad screening of synthetically introduced acyl chains. However, an additional challenge is that CDAs are usually produced on solid media only and in very low quantities.^[13a]^

In conclusion, CDAs from *S. violaceoruber* are deacylated in co‐culture with *Streptomyces* sp. MG7‐G1, in particular when the concentration of Ca^2+^ ions is high suggesting that *Streptomyces* sp. MG7‐G1 inactivates CDAs.

Co‐cultivation of microorganisms – mimicking natural conditions – in combination with mass spectrometric screening is an established useful method for the discovery of new metabolites that are only produced when organisms grow together.[^1^, ^5^] Our example of the *Streptomyces* sp. MG7‐G1/*S. violaceoruber* co‐culture nicely demonstrates that this approach not only helps to identify novel secondary metabolites from so‐called silent gene clusters, but that it can also help to reveal unexpected enzymatic functions.

Future experiments are needed to identify and characterise the CDA deacylase. This will allow to study in detail both its biological function and to evaluate its potential use to generate acyl‐chain‐modified CDAs with optimized pharmaceutical properties.

## Experimental Section

**General experimental procedures**: Chemicals were purchased from Sigma and Carl Roth. HPLC of droplet samples and agar exudate was performed with either an LTQ (Thermo Fisher) equipped with an electrospray ionization (ESI) source operated in the positive mode connected to a Waters Acquity UPLC system (Waters GmbH, Eschborn; Germany) or a Thermo Fisher Orbitrap XL mass spectrometer (Thermo Fisher) fitted with a HESI‐II ion source operated in the positive mode connected to a Dionex Ultimate 3000 UHPLC system (Thermo Fisher).

**Strains and cultivation conditions**: *Streptomyces violaceoruber* DSM 40783 (strain designation A3(2) and *Streptomyes* sp. MG7‐G1 ATCC31860 were cultivated on soy flour mannitol (SFM) agar plates. For the SFM medium (soy flour 20 g L^−1^, mannitol 10 g L^−1^, agar 15 g L^−1^) either tap water or doubly distilled water was used. Drops (1 μL) of spore suspensions^[33]^ of *S. violaceoruber* and *Streptomyes* sp. MG7‐G1 were spotted either alone (single culture) or alternately (co‐culture) in the shape of a cross onto the agar plates. The agar plates were incubated at 28 °C (Figure S1).

**Collection of droplets**: Droplets of *S. violaceoruber* cultivated in single‐ and co‐culture with *Streptomyes* sp. MG7‐G1 were collected with pulled glass capillaries (Ø_in_ 0.56 mm, Hilgenberg, Malsfeld, Germany) on days 7 to 24 (mainly days 12–15). Droplets were stored at −20 °C until further analysis by LC‐MS.

**Extraction of agar medium**: CDAs and daCDAs in agar medium (9 cm diameter) were analysed by collecting the exudate from 10–20 d old agar plates of co‐cultures and single cultures after freezing and thawing.^[13a]^ The samples were acidified to pH 1–2 with 1 N HCl, vortexed for 0.5 min, centrifuged at 7233 *g* for 1 min and analysed by LC‐MS.

**Secondary metabolite profiling by LC‐MS**: For droplet analysis by LCMS‐ESI 2–50 μL of a sample were analysed either by an LTQ or an LTQ Orbitrap XL mass spectrometer (Thermo Fisher). An Accucore RP‐MS column (100 mm×2.1 mm, 2.6 μm, Thermo Fisher) was used for programmed elution with H_2_O+0.1 % AcOH (solvent A) and MeOH+0.1 % AcOH (solvent B) at a flow rate of 0.3 mL/min. HPLC‐programme: 30 % B for 0.5 min, from 30 to 100 % B in 7.5 min, 4 min 100 % B, from 100 % to 30 % B in 0.5 min, re‐equilibration at 30 % B for 4 min. ESI‐MS/MS experiments were performed fragmenting the quasimolecular ions at 35–55 % energy.

**Determination of molecular composition**: The molecular formulas of CDAs and daCDAs were obtained using an Orbitrap XL mass spectrometer for HR‐HESI‐MS measurements at the 100 000 resolution setting using the positive calibration mixture as lock masses. MS/MS measurements were performed at 35–55 % collision energy at 7500 to 100 000 resolution.

**Influence of calcium ions**: Sterilised SFM media, which were prepared with ddH_2_O, were supplemented with CaCl_2_ to final concentrations of 1, 2, 10, and 20 mM. Co‐cultures were grown on these media as described above. Exudate was collected after 8–20 days of growth.^[13a]^ Samples were analysed by LC‐MS. The peak areas of CDAs were compared to those of daCDAs.

## Conflict of interest

The authors declare no conflict of interest.

## Supporting information

As a service to our authors and readers, this journal provides supporting information supplied by the authors. Such materials are peer reviewed and may be re‐organized for online delivery, but are not copy‐edited or typeset. Technical support issues arising from supporting information (other than missing files) should be addressed to the authors.

Supplementary
