# Supplementary material for: Deacylation of Calcium‐Dependent Antibiotics from Streptomyces violaceoruber in Co‐culture with Streptomyces sp. MG7‐G1
Source: Chembiochem. 2020 Jul 20;21(21):3151–7. doi: 10.1002/cbic.202000404 (PMC7689815; doi:10.1002/cbic.202000404)
Supplement: Supplementary file 1 — Supplementary [file CBIC-21-3151-s001.pdf]

ChemBioChem

Supporting Information

Deacylation of Calcium-Dependent Antibiotics from *Streptomyces violaceoruber* in Co-culture with *Streptomyces* sp. MG7-G1

Kathrin Schindl, Deepika Sharma, and Dieter Spiteller*

Co-culture of *S. violaceoruber* and *Streptomyces* sp. MG7-G1 2
HR-ESI-MS and HR-ESI-MS/MS of daCDA 3a (**5**) 3
HR-ESI-MS and HR-ESI-MS/MS of daCDA 3b (**6**) 5
HR-ESI-MS and HR-ESI-MS/MS of daCDA 4a (**7**) 7
HR-ESI-MS and HR-ESI-MS/MS of daCDA 4b (**8**) 9
HR-ESI-MS and HR-ESI-MS/MS of CDA 3a (**1**) 11
HR-ESI-MS and HR-ESI-MS/MS of CDA 3b (**2**) 13
HR-ESI-MS and HR-ESI-MS/MS of CDA 4a (**3**) 15
HR-ESI-MS and HR-ESI-MS/MS of CDA 4b (**4**) 17
CDAs and daCDAs in droplets from *Streptomyces violaceoruber* in single culture and co-culture with *Streptomyces* sp. MG7-G1 19
Transport of compounds from the medium (fluorescein) into the droplets 20
LC-MS ion traces for CDAs and daCDAs of *Streptomyces* sp. MG7-G1 exudate 21

Co-culture of *S. violaceoruber* and *Streptomyces* sp. MG7-G1

A

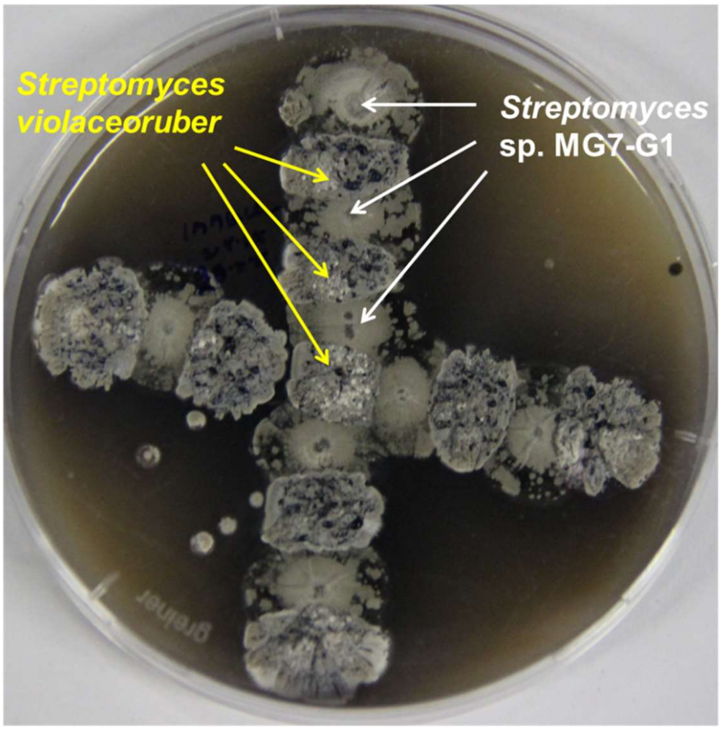

B

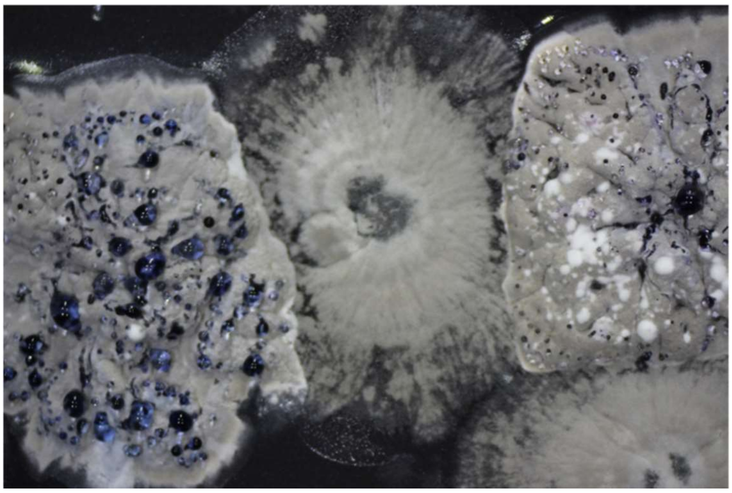

Figure S1:

A) Co-culture of *S. violaceoruber* and *Streptomyces* sp. MG7-G1 on SFM agar plates. *S. violaceoruber* and *Streptomyces* sp. MG7-G1 colonies alternate shaping a cross on the SFM agar.

B) Detailed view of *S. violaceoruber* and *Streptomyces* sp. MG7-G1 colonies growing together (18 d). *S. violaceoruber* produces blue droplets on top of its aerial mycelium.

HR-ESI-MS and HR-ESI-MS/MS of daCDA 3a (5)

daCDA 3a (**5**) $[M+H]^+$: retention time: 7.0 min, $[M+H]^+$: $C_{60}H_{69}N_{14}O_{24}$ observed: 1369.46073, calculated: 1369.46037, Δ: 0.3 ppm.

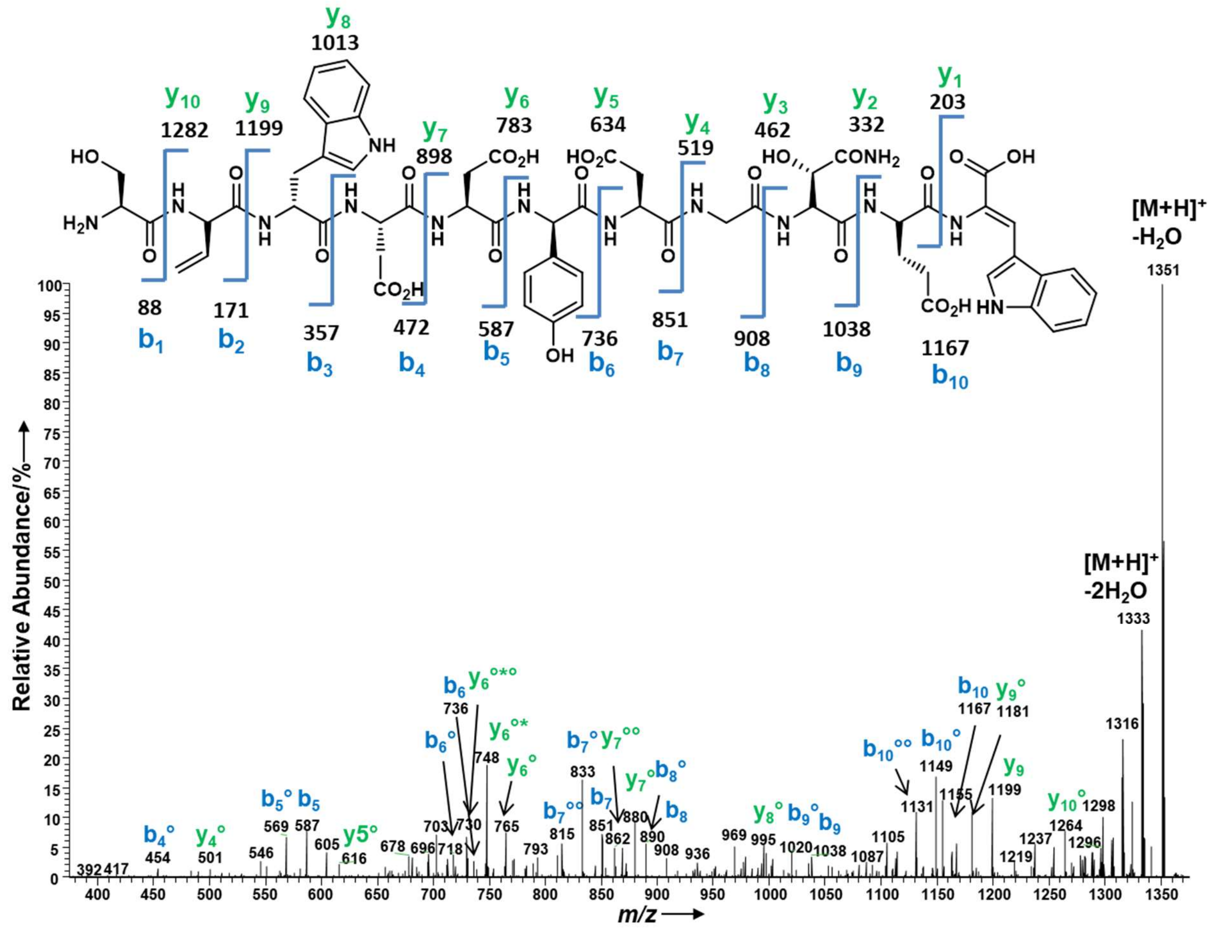

Figure S2: MS/MS fragmentation of the quasimolecular ion $[M+H]^+$ of daCDA4a (**5**). After lactone ring opening the linear $[M+H]^+$ m/z 1369 forms the y- and b-ions. Ions resulting from an additional loss of H_2O are indicated by ° and those resulting from a loss of NH_3 by *.

Table S1: MS/MS fragments of the quasimolecular ion $[M+H]^+$1369 of daCDA 3a (5)

ion	formula	m/z	Δppm
$M+H^+-H_2O$	$C_{60}H_{67}O_{23}N_{14}$	1351.44861	0.8
$M+H^+-2H_2O$	$C_{60}H_{65}O_{22}N_{14}$	1333.43811	0.8
b10	$C_{49}H_{59}O_{22}N_{12}$	1167.38538	0.6
b10-H_2O	$C_{49}H_{57}O_{21}N_{12}$	1149.37512	0.4
b10-2H_2O	$C_{49}H_{55}O_{20}N_{12}$	1131.36462	0.3
b9	$C_{44}H_{52}O_{19}N_{11}$	1038.34351	0.0
b9-H_2O	$C_{44}H_{50}O_{18}N_{11}$	1020.33301	0.0
b8	$C_{40}H_{46}O_{16}N_9$	908.30591	0.2
b8-H_2O	$C_{40}H_{44}O_{15}N_9$	890.29474	0.5
b8-2H_2O	$C_{40}H_{42}O_{14}N_9$	872.28473	0.2
b7	$C_{38}H_{43}O_{15}N_8$	851.28430	0.1
b7-H_2O	$C_{38}H_{41}O_{14}N_8$	833.27338	0.4
b7-2H_2O	$C_{38}H_{39}O_{13}N_8$	815.26270	0.5
b6	$C_{34}H_{38}O_{12}N_7$	736.25714	0.2
b6-H_2O	$C_{34}H_{36}O_{11}N_7$	718.24707	0.5
b6-2H_2O	$C_{34}H_{34}O_{10}N_7$	700.23651	0.5
b5	$C_{26}H_{31}O_{10}N_6$	587.20935	0.5
b5-H_2O	$C_{26}H_{29}O_9N_6$	569.19904	0.0
b4	$C_{22}H_{26}O_7N_5$	472.18259	0.2
b4-H_2O	$C_{22}H_{24}O_6N_5$	454.17160	1.1
y10	$C_{57}H_{64}O_{22}N_{13}$	1282.42725	0.9
y10-H_2O	$C_{57}H_{62}O_{21}N_{13}$	1264.41699	0.6
y9	$C_{53}H_{59}O_{21}N_{12}$	1199.39075	0.4
y9-H_2O	$C_{53}H_{57}O_{20}N_{12}$	1181.38000	0.6
y8	$C_{42}H_{49}O_{20}N_{10}$	1013.31201	0.1
y8- H_2O	$C_{42}H_{47}O_{19}N_{10}$	995.30103	0.3
y7-H_2O	$C_{38}H_{42}O_{16}N_9$	880.27411	0.3
y6	$C_{34}H_{39}O_{14}N_8$	783.25800	0.0
y6-H_2O	$C_{34}H_{37}O_{13}N_8$	765.24731	0.2
y6-H_2O-NH_3	$C_{34}H_{34}O_{13}N_7$	748.22071	0.3
y6-H_2O-NH_3-H_2O	$C_{34}H_{32}O_{12}N_7$	730.21025	0.1

HR-ESI-MS and HR-ESI-MS/MS of daCDA 3b (6)

daCDA 3b (**6**) $[M+H]^+$: retention time: 6.6 min, $[M+H]^+$: $C_{60}H_{71}N_{14}O_{24}$ observed: 1371.47613, calculated: 1371.47602, Δ: 0.1 ppm.

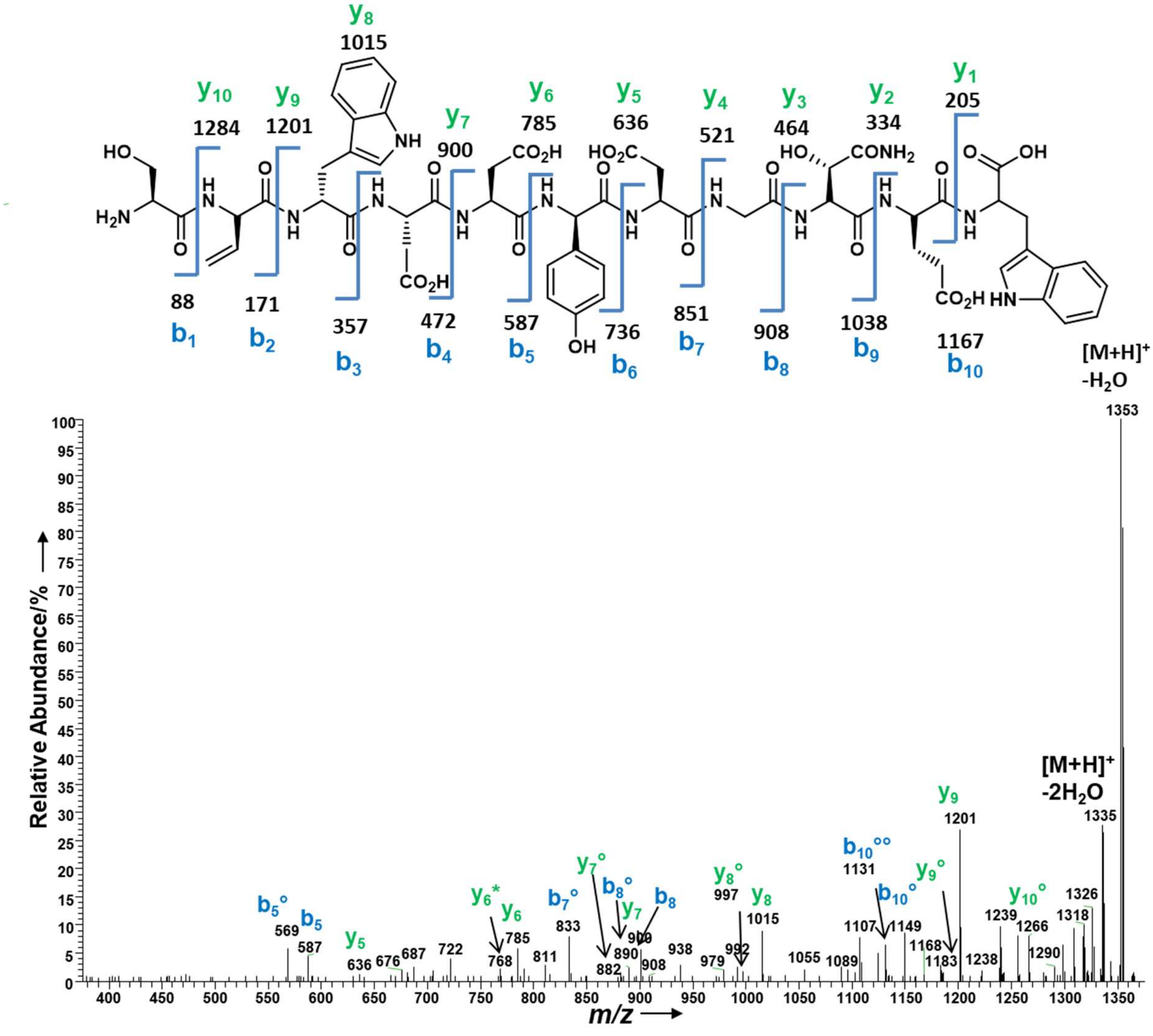

Figure S3: MS/MS fragmentation of the quasimolecular ion $[M+H]^+$ of daCDA 3b (**6**). After lactone ring opening the linear $[M+H]^+$ m/z 1371 forms the y- and b-ions. Ions resulting from an additional loss of H_2O are indicated by ° and those resulting from a loss of NH_3 by *.

Table S2: MS/MS fragments of the quasimolecular ion $[M+H]^+$1371 of daCDA 3b (6)

ion	formula	m/z	Δppm
$M+H^+-H_2O$	$C_{60}H_{69}O_{23}N_{14}$	1353.46399	0.9
$M+H^+-2H_2O$	$C_{60}H_{67}O_{22}N_{14}$	1335.45464	0.2
b10-H_2O	$C_{49}H_{57}O_{21}N_{12}$	1149.37379	1.6
b10-2H_2O	$C_{49}H_{55}O_{20}N_{12}$	1131.36243	2.3
b8-H_2O	$C_{40}H_{44}O_{15}N_9$	890.29373	1.6
b7-H_2O	$C_{38}H_{41}O_{14}N_8$	833.27303	0.8
b6- H_2O	$C_{34}H_{36}O_{11}N_7$	718.24689	0.2
b5	$C_{26}H_{31}O_{10}N_6$	587.20928	0.6
b5-H_2O	$C_{26}H_{29}O_9N_6$	569.19913	0.1
y10-H_2O	$C_{57}H_{64}O_{21}N_{13}$	1266.43186	1.2
y9	$C_{53}H_{61}O_{21}N_{12}$	1201.40580	0.9
y9-H_2O	$C_{53}H_{59}O_{20}N_{12}$	1183.39942	2.6
y8	$C_{42}H_{51}O_{20}N_{10}$	1015.32626	1.3
y8- H_2O	$C_{42}H_{49}O_{19}N_{10}$	997.31818	1.2
y7-H_2O	$C_{38}H_{44}O_{16}N_9$	882.28711	3.4
y6	$C_{34}H_{41}O_{14}N_8$	785.27351	0.2
y6-H_2O-NH_3	$C_{34}H_{36}O_{13}N_7$	750.23468	2.5

HR-ESI-MS and HR-ESI-MS/MS of daCDA 4a (7)

daCDA 4a (**7**) $[M+H]^+$: retention time: 7.0 min, $[M+H]^+$: $C_{61}H_{71}N_{14}O_{24}$ observed: 1383.47599, calculated: 1383.47571, Δ: 0.1 ppm.

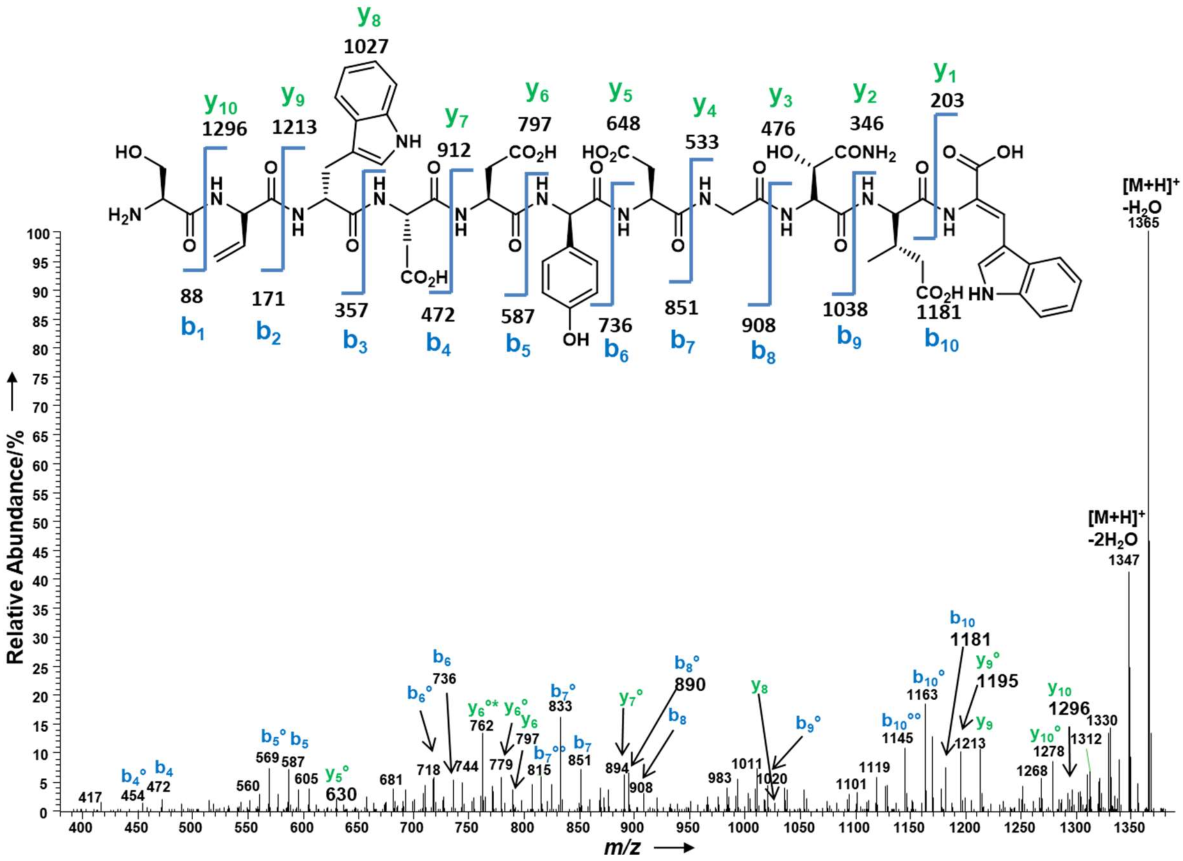

Figure S4: MS/MS fragmentation of the quasimolecular ion $[M+H]^+$ of daCDA 4a (**7**). After lactone ring opening the linear $[M+H]^+$ m/z 1383 forms the y- and b-ions. Ions resulting from an additional loss of H_2O are indicated by ° and those resulting from a loss of NH_3 by *.

Table S3: MS/MS fragments of the quasimolecular ion $[M+H]^+$1383 of daCDA 4a (7)

ion	formula	m/z	Δppm
$M+H^+-H_2O$	$C_{61}H_{69}O_{23}N_{14}$	1365.46378	1.0
$M+H^+-2H_2O$	$C_{61}H_{67}O_{22}N_{14}$	1347.45349	1.0
b10	$C_{50}H_{61}O_{22}N_{12}$	1181.40112	0.6
b10-H_2O	$C_{50}H_{59}O_{21}N_{12}$	1163.39026	0.8
b10-2H_2O	$C_{50}H_{57}O_{20}N_{12}$	1145.38013	0.5
b9	$C_{44}H_{52}O_{19}N_{11}$	1038.34241	1.1
b9-H_2O	$C_{44}H_{50}O_{18}N_{11}$	1020.33203	0.9
b8	$C_{40}H_{46}O_{16}N_9$	908.30566	0.1
b8-H_2O	$C_{40}H_{44}O_{15}N_9$	890.29462	0.6
b8-2H_2O	$C_{40}H_{42}O_{14}N_9$	872.28387	0.8
b7	$C_{38}H_{43}O_{15}N_8$	851.28400	0.3
b7-H_2O	$C_{38}H_{41}O_{14}N_8$	833.27325	0.5
b7-2H_2O	$C_{38}H_{39}O_{13}N_8$	815.26288	0.3
b6	$C_{34}H_{38}O_{12}N_7$	736.25690	0.5
b6- H_2O	$C_{34}H_{36}O_{11}N_7$	718.24646	0.4
b6-2H_2O	$C_{34}H_{34}O_{10}N_7$	700.23566	0.7
b5	$C_{26}H_{31}O_{10}N_6$	587.20923	0.7
b5-H_2O	$C_{26}H_{29}O_9N_6$	569.19879	0.5
b4	$C_{22}H_{26}O_7N_5$	472.18250	0.4
b4-H_2O	$C_{22}H_{24}O_6N_5$	454.17221	0.2
y10	$C_{58}H_{66}O_{22}N_{13}$	1296.44263	1.1
y10-H_2O	$C_{58}H_{64}O_{21}N_{13}$	1278.43213	1.0
y9	$C_{54}H_{61}O_{21}N_{12}$	1213.40588	0.8
y9-H_2O	$C_{54}H_{59}O_{20}N_{12}$	1195.39539	0.8
y8	$C_{43}H_{48}O_{20}N_9$	1013.31201	0.1
y7-H_2O	$C_{39}H_{44}O_{16}N_9$	894.28949	0.6
y6	$C_{35}H_{41}O_{14}N_8$	797.27338	0.4
y6-H_2O	$C_{35}H_{39}O_{13}N_8$	779.26282	0.4
y6-H_2O-NH_3	$C_{35}H_{36}O_{13}N_7$	762.23632	0.3
y5-H2O	$C_{27}H_{32}O_{11}N_7$	630.21539	0.1

HR-ESI-MS and HR-ESI-MS/MS of daCDA 4b (8)

daCDA 4b (**8**) $[M+H]^+$: retention time: 6.6 min, $[M+H]^+$: $C_{61}H_{73}N_{14}O_{24}$ observed: 1385.49156, calculated: 1385.49167, Δ: 0.1 ppm.

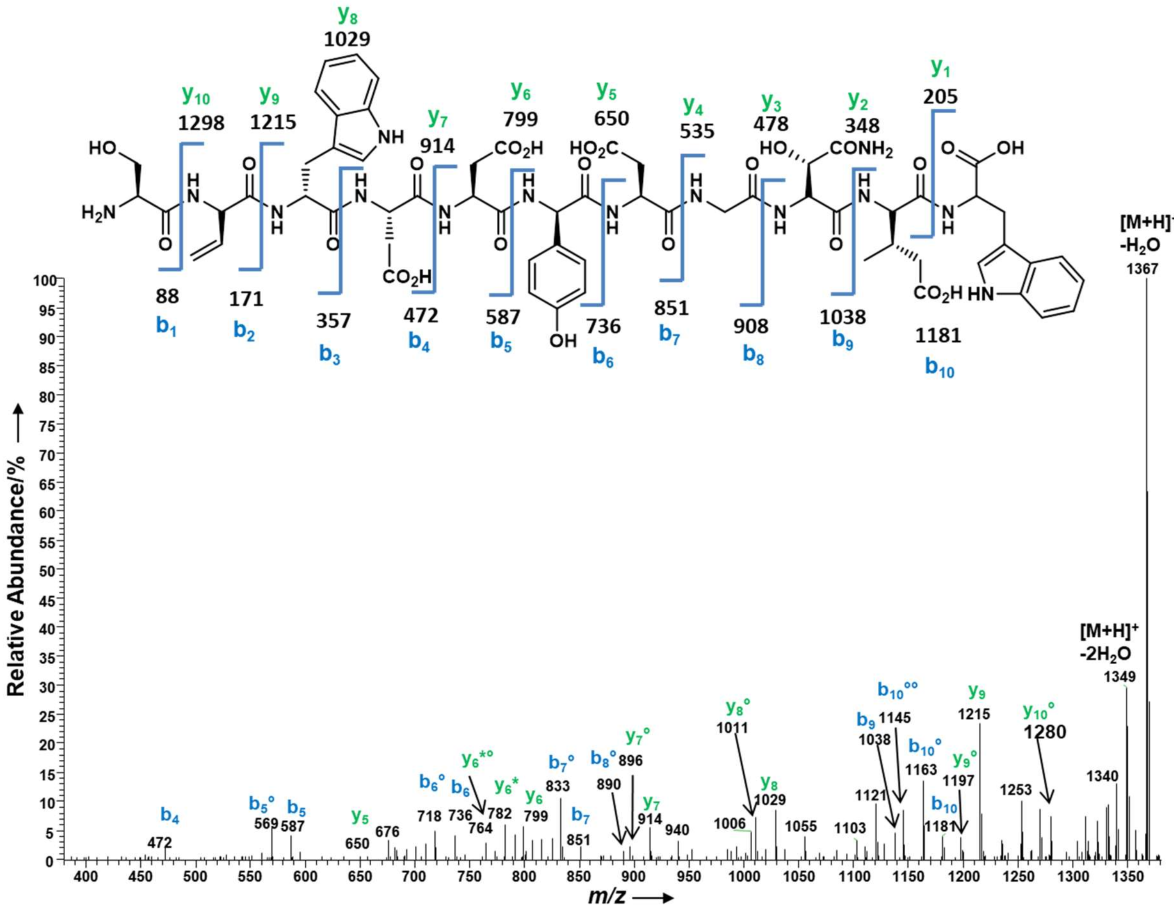

Figure S5: MS/MS fragmentation of the quasimolecular ion $[M+H]^+$ of daCDA 4b (**8**). After lactone ring opening the linear $[M+H]^+$ m/z 1385 forms the y- and b-ions. Ions resulting from an additional loss of H_2O are indicated by ° and those resulting from a loss of NH_3 by *.

Table S4: MS/MS fragments of the quasimolecular ion $[M+H]^+$1385 of daCDA 4b (8)

ion	formula	m/z	Δppm
$M+H^+-H_2O$	$C_{61}H_{71}O_{23}N_{14}$	1367.47961	1.1
$M+H^+-2H_2O$	$C_{61}H_{69}O_{22}N_{14}$	1349.46920	1.0
b10	$C_{50}H_{61}O_{22}N_{12}$	1181.40126	0.5
b10-H_2O	$C_{50}H_{59}O_{21}N_{12}$	1163.39038	0.7
b10-2H_2O	$C_{50}H_{57}O_{20}N_{12}$	1145.37978	0.8
b9-H_2O	$C_{44}H_{50}O_{18}N_{11}$	1020.33144	1.5
b8-H_2O	$C_{40}H_{44}O_{15}N_9$	890.29489	0.3
b7	$C_{38}H_{43}O_{15}N_8$	851.28406	0.2
b7-H_2O	$C_{38}H_{41}O_{14}N_8$	833.27315	0.6
b7-2H_2O	$C_{38}H_{39}O_{13}N_8$	815.26321	0.1
b6	$C_{34}H_{38}O_{12}N_7$	736.25668	0.8
b6- H_2O	$C_{34}H_{36}O_{11}N_7$	718.24637	0.5
b5	$C_{26}H_{31}O_{10}N_6$	587.20930	0.5
b5-H_2O	$C_{26}H_{29}O_9N_6$	569.19867	0.7
b4	$C_{22}H_{26}O_7N_5$	472.18220	1.0
y10-H_2O	$C_{58}H_{66}O_{21}N_{13}$	1280.44777	1.0
y9	$C_{54}H_{63}O_{21}N_{12}$	1215.42159	0.8
y8	$C_{43}H_{53}O_{20}N_{10}$	1029.34237	0.8
y8-H_2O	$C_{43}H_{51}O_{19}N_{10}$	1111.33162	1.0
y7	$C_{39}H_{48}O_{17}N_9$	914.31554	0.8
y7-H_2O	$C_{39}H_{46}O_{16}N_9$	896.30552	0.2
y6	$C_{35}H_{43}O_{14}N_8$	799.28915	0.2
y6-NH_3	$C_{35}H_{40}O_{14}N_7$	782.30552	1.0
y6-NH_3-H_2O	$C_{35}H_{38}O_{13}N_8$	764.25229	0.1

HR-ESI-MS and HR-ESI-MS/MS of CDA 3a (1)

CDA 3a (**1**) $[M+H]^+$: retention time: 8.5 min, $C_{66}H_{77}N_{14}O_{26}$ observed: 1481.51306, calculated: 1481.51323, Δ: 0.3 ppm.

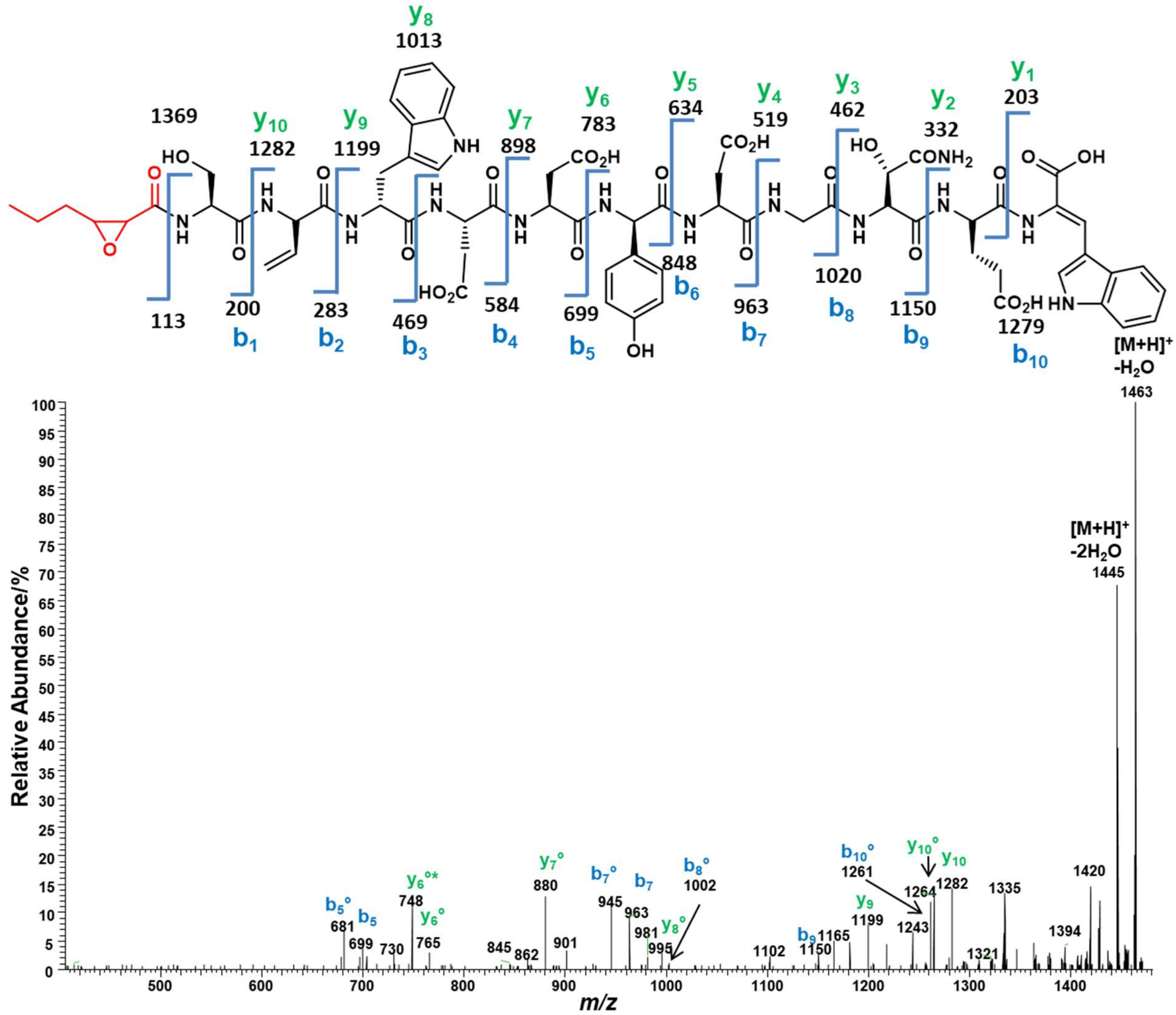

Figure S6: MS/MS fragmentation of the quasimolecular ion $[M+H]^+$ of CDA 3a (**1**). After lactone ring opening the linear $[M+H]^+$ m/z 1481 forms the y- and b-ions. Ions resulting from an additional loss of H_2O are indicated by ° and those resulting from a loss of NH_3 by *.

Table S5: MS/MS fragments of the quasimolecular ion $[M+H]^{+}$1481 of CDA 3a (1)

ions	formula	m/z	Δppm
$M+H^{+}-H_2O$	$C_{66}H_{75}O_{25}N_{14}$	1463.49974	1.7
$M+H^{+}-2H_2O$	$C_{66}H_{73}O_{24}N_{14}$	1445.48955	1.5
b10-H_2O	$C_{55}H_{65}O_{23}N_{12}$	1261.42519	2.2
b9	$C_{50}H_{60}O_{21}N_{11}$	1150.39662	0.6
b8-H_2O	$C_{46}H_{52}O_{17}N_9$	1002.16274	
b7	$C_{44}H_{51}O_{17}N_8$	963.33511	1.6
b7-H_2O	$C_{44}H_{49}O_{16}N_8$	945.32632	0.2
b5	$C_{32}H_{39}O_{12}N_6$	699.26268	0.9
b4-H_2O	$C_{28}H_{32}O_8N_5$	566.22356	1.7
y10	$C_{57}H_{64}O_{22}N_{13}$	1282.42684	1.2
y10-H_2O	$C_{57}H_{62}O_{21}N_{13}$	1264.41683	0.7
y9	$C_{53}H_{59}O_{21}N_{12}$	1199.39041	0.7
y7-H_2O	$C_{38}H_{42}O_{16}N_9$	880.27318	1.4
y6-H_2O	$C_{34}H_{37}O_{13}N_8$	765.24799	1.1
y6-H_2O-NH_3	$C_{34}H_{34}O_{13}N_7$	748.21958	1.8

HR-ESI-MS and HR-ESI-MS/MS of CDA 3b (2)

CDA 3b (**2**) $[M+H]^+$: retention time: 8.4 min, $C_{66}H_{79}N_{14}O_{26}$ observed: 1483.52832, calculated: 1483.52845, Δ: 0.1 ppm.

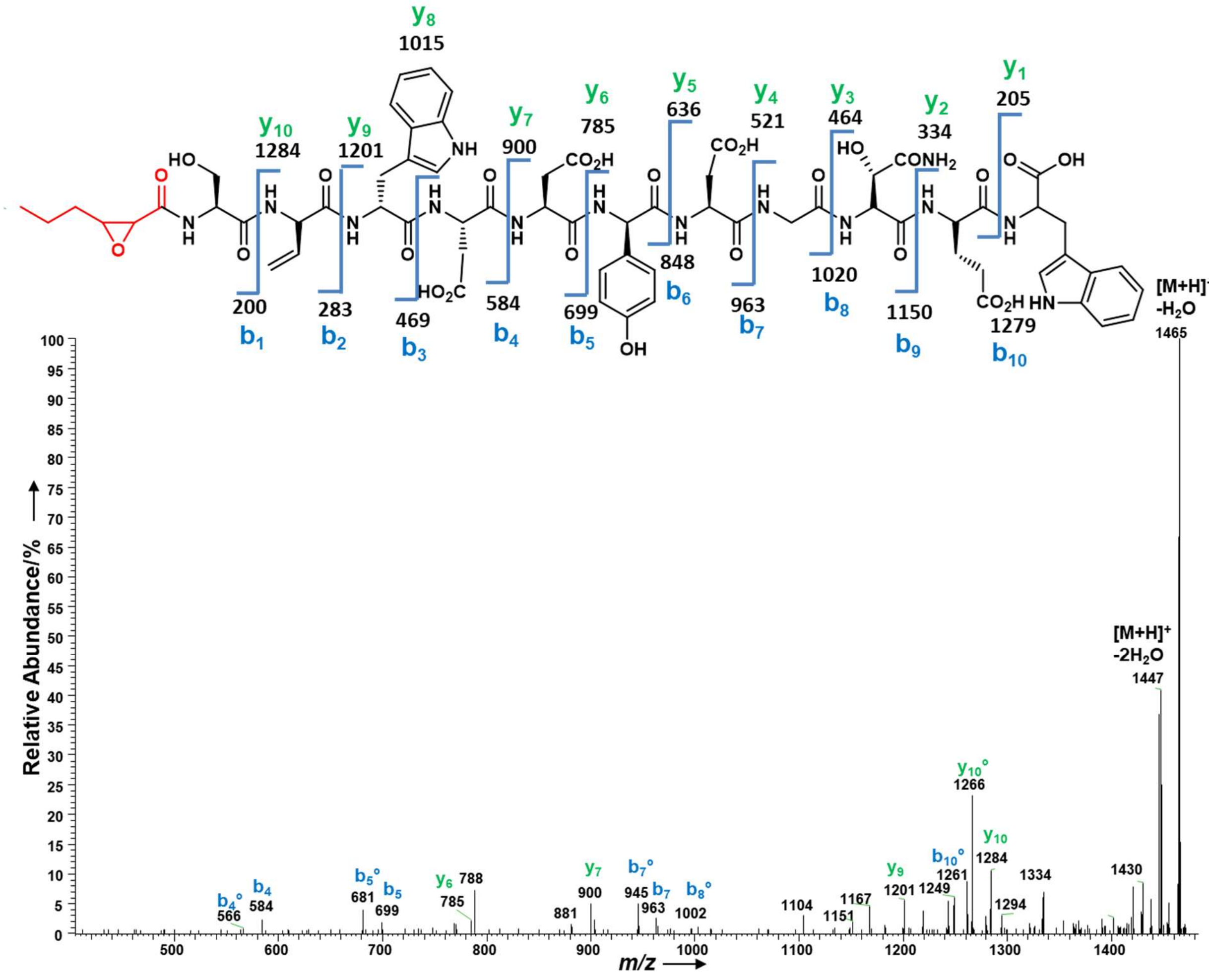

Figure S7: MS/MS fragmentation of the quasimolecular ion $[M+H]^+$ of CDA 3b (**2**). After lactone ring opening the linear $[M+H]^+$ m/z 1483 forms the y- and b-ions. Ions resulting from an additional loss of H_2O are indicated by ° and those resulting from a loss of NH_3 by *.

Table S6: MS/MS fragments of the quasimolecular ion $[M+H]^+$1483 of CDA 3b (2)

ions	formula	m/z	Δppm
$M+H^+-H_2O$	$C_{66}H_{77}O_{25}N_{14}$	1465.51522	1.8
$M+H^+-2H_2O$	$C_{66}H_{75}O_{24}N_{14}$	1447.50372	2.5
b10	$C_{55}H_{67}O_{24}N_{12}$	1279.43579	2.1
b10-H_2O	$C_{55}H_{65}O_{23}N_{12}$	1261.42567	1.9
b10-2H_2O	$C_{55}H_{63}O_{22}N_{12}$	1243.41587	1.3
b9-H_2O	$C_{50}H_{58}O_{20}N_{11}$	1132.38232	2.7
b7	$C_{44}H_{51}O_{17}N_8$	963.33746	0.8
b7-H_2O	$C_{44}H_{49}O_{16}N_8$	945.32486	1.3
b5-H_2O	$C_{32}H_{37}O_{11}N_6$	681.25098	0.5
b4	$C_{28}H_{34}O_9N_5$	584.23480	0.5
y10-H_2O	$C_{57}H_{64}O_{21}N_{13}$	1266.43135	1.6
y9	$C_{53}H_{61}O_{21}N_{12}$	1201.40485	1.7
y9-H_2O	$C_{53}H_{59}O_{20}N_{12}$	1183.39648	0.2
y8	$C_{42}H_{51}O_{19}N_9$	1015.32684	0.7
y7	$C_{38}H_{46}O_{17}N_9$	900.29904	1.8

HR-ESI-MS and HR-ESI-MS/MS of CDA 4a (3)

CDA 4a (**3**) $[M+H]^+$: retention time: 8.6 min, $[M+H]^+$ $C_{67}H_{79}N_{14}O_{26}$ observed: 1495.52841, calculated: 1495.52845, Δ: 0.0 ppm.

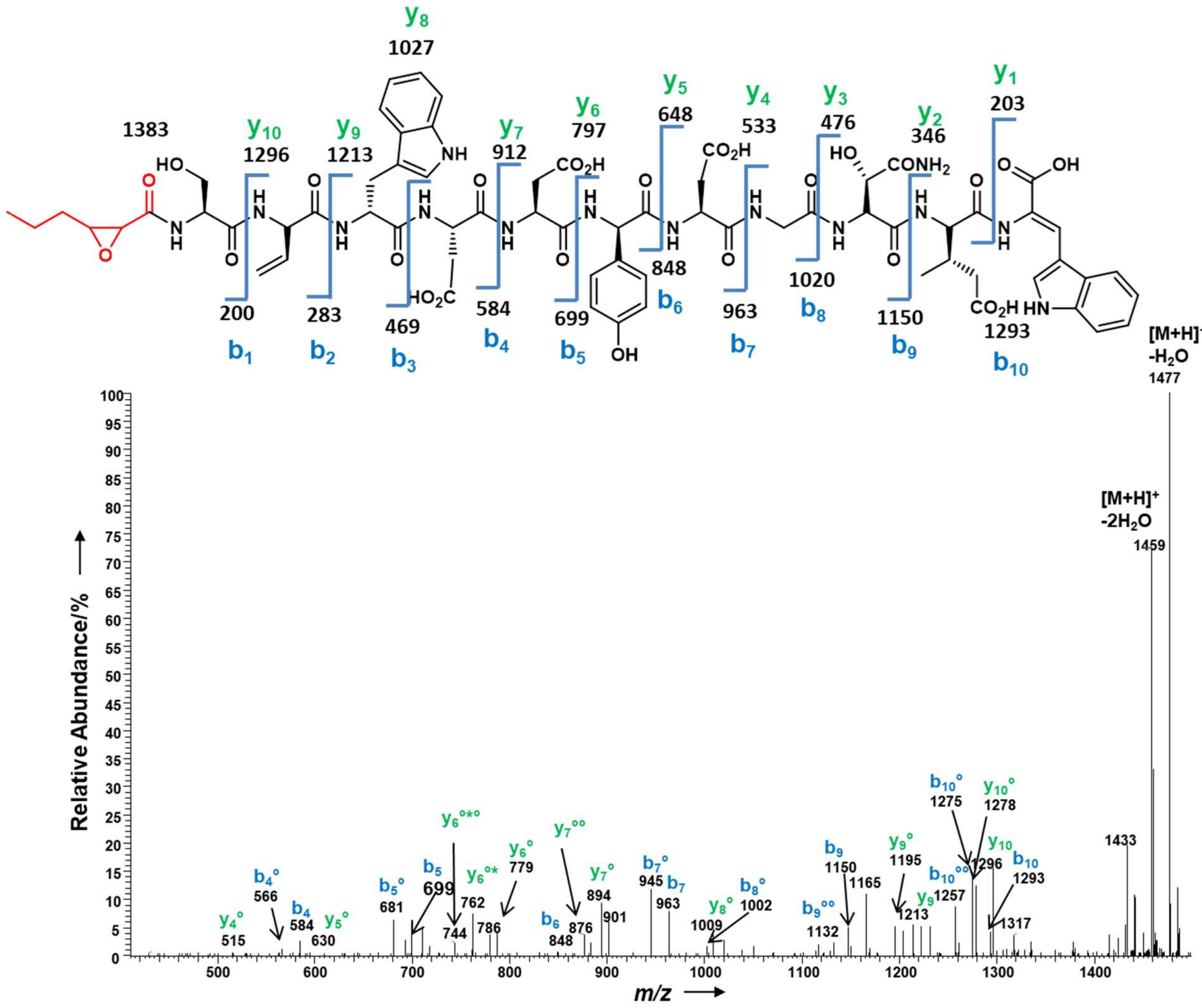

Figure S8: MS/MS fragmentation of the quasimolecular ion $[M+H]^+$ of CDA 4a (**3**). After lactone ring opening the linear $[M+H]^+$ m/z 1495 forms the y- and b-ions. Ions resulting from an additional loss of H_2O are indicated by ° and those resulting from a loss of NH_3 by *.

Table S7: MS/MS fragments of the quasimolecular ion $[M+H]^+$1495 of CDA 4a (3)

ions	formula	m/z	Δppm
$M+H^+-H_2O$	$C_{67}H_{77}O_{25}N_{14}$	1477.51538	0.0
$M+H^+-2H_2O$	$C_{67}H_{75}O_{24}N_{14}$	1459.50445	0.3
b10	$C_{56}H_{69}O_{24}N_{12}$	1293.45244	1.3
b10-H_2O	$C_{56}H_{67}O_{23}N_{12}$	1275.44229	1.0
b10-2H_2O	$C_{56}H_{65}O_{22}N_{12}$	1257.43159	0.2
b9	$C_{50}H_{60}O_{21}N_{11}$	1150.39561	0.3
b9-H_2O	$C_{50}H_{58}O_{20}N_{11}$	1132.38303	2.1
b8	$C_{46}H_{54}O_{18}N_9$	1020.35634	1.8
b8-H_2O	$C_{46}H_{52}O_{17}N_9$	1002.34778	0.2
b7	$C_{44}H_{51}O_{17}N_8$	963.33571	1.0
b7-H_2O	$C_{44}H_{49}O_{16}N_8$	945.32516	1.0
b7-2H_2O	$C_{44}H_{47}O_{15}N_8$	927.31330	2.4
b5	$C_{32}H_{39}O_{12}N_6$	699.26211	0.0
b5-H_2O	$C_{32}H_{37}O_{11}N_6$	681.25091	0.6
b4	$C_{28}H_{34}O_9N_5$	584.23431	0.8
b4-H_2O	$C_{28}H_{32}O_8N_5$	566.22430	0.2
y10	$C_{58}H_{66}O_{22}N_{13}$	1296.44159	1.9
y10-H_2O	$C_{58}H_{64}O_{21}N_{13}$	1278.43148	1.5
y9	$C_{54}H_{61}O_{21}N_{12}$	1213.40448	2.0
y9-H_2O	$C_{54}H_{59}O_{20}N_{12}$	1195.39556	0.6
y8-H_2O	$C_{43}H_{49}O_{19}N_9$	1009.31626	0.7
y7-H_2O	$C_{39}H_{44}O_{16}N_9$	894.28912	1.0
y6	$C_{35}H_{41}O_{14}N_8$	797.27356	0.1
y6-H_2O	$C_{35}H_{39}O_{13}N_8$	779.26131	2.3
y6-H_2O-NH_3	$C_{35}H_{36}O_{13}N_7$	762.23630	0.3
y6-H_2O-NH_3-H_2O	$C_{35}H_{34}O_{12}N_7$	744.22652	0.7

HR-ESI-MS and HR-ESI-MS/MS of CDA 4b (4)

CDA 4b (**4**) $[M+H]^+$: retention time: 8.5 min, $[M+H]^+$: $C_{67}H_{81}N_{14}O_{26}$ observed: 1497.54468, calculated: 1497.54410, Δ: 0.4 ppm.

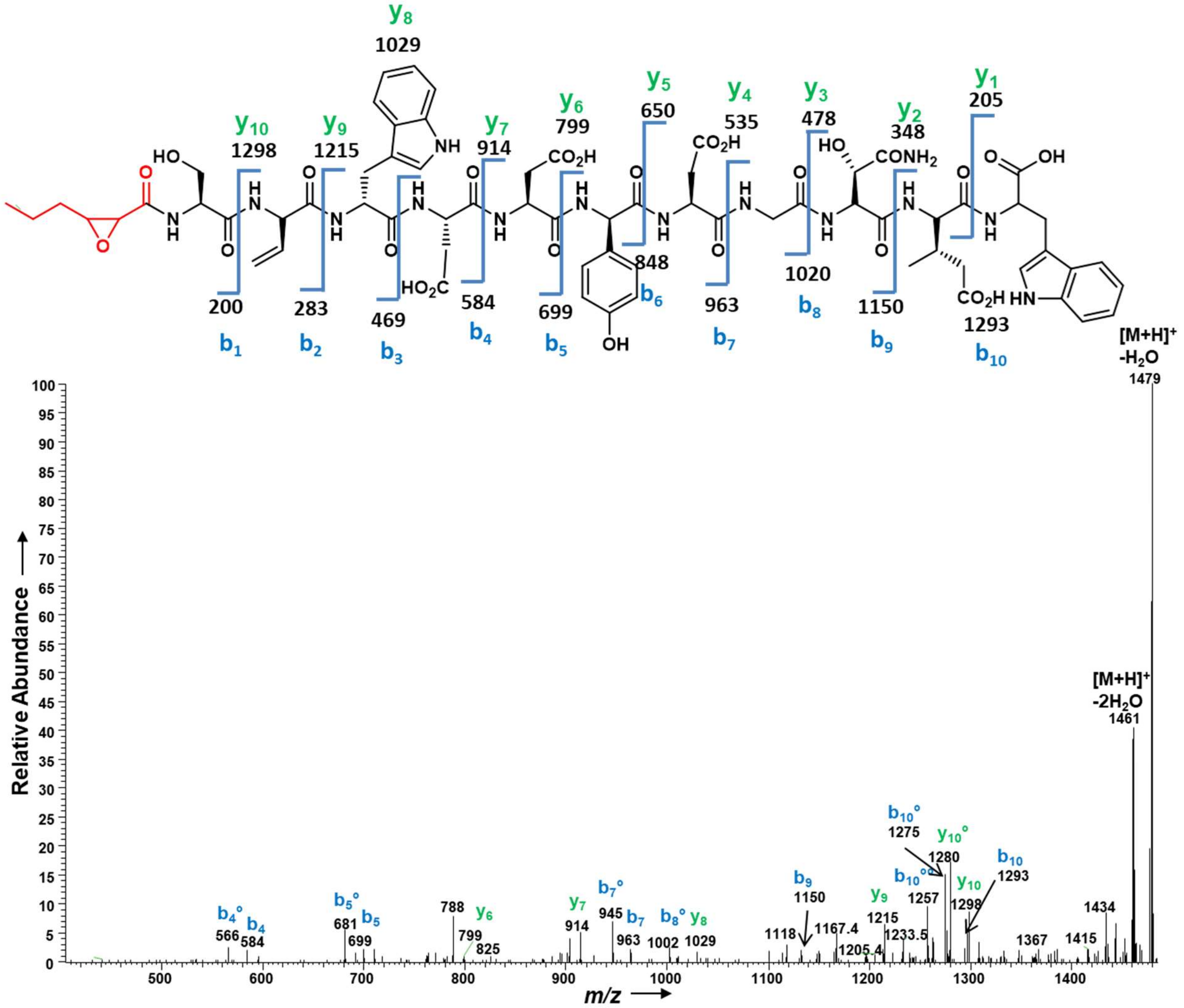

Figure S9: MS/MS fragmentation of the quasimolecular ion $[M+H]^+$ of CDA 4b (**4**). After lactone ring opening the linear $[M+H]^+$ m/z 1497 forms the typical y- and b-ions. Ions resulting from an additional loss of H_2O are indicated by ° and those resulting from a loss of NH_3 by *.

Table S8: MS/MS fragments of the quasimolecular ion $[M+H]^+$1497 of CDA 4b (4)

ions	formula	m/z	Δppm
$M+H^+-H_2O$	$C_{67}H_{79}O_{25}N_{14}$	1479.53149	1.4
$M+H^+-2H_2O$	$C_{67}H_{77}O_{24}N_{14}$	1459.50445	1.1
b10	$C_{56}H_{69}O_{24}N_{12}$	1293.45018	3.1
b10-H_2O	$C_{56}H_{67}O_{23}N_{12}$	1275.44211	1.2
b10-2H_2O	$C_{56}H_{65}O_{22}N_{12}$	1257.43091	1.7
b9-H_2O	$C_{50}H_{58}O_{20}N_{11}$	1132.38517	0.2
b8	$C_{46}H_{54}O_{18}N_{9}$	1020.36011	1.9
b8-H_2O	$C_{46}H_{52}O_{17}N_{9}$	1002.34637	1.2
b7	$C_{44}H_{51}O_{17}N_{8}$	963.33585	0.9
b7-H_2O	$C_{44}H_{49}O_{16}N_{8}$	945.32537	0.8
b6-H_2O	$C_{40}H_{44}O_{13}N_{7}$	830.29749	2.0
b5	$C_{32}H_{39}O_{12}N_{6}$	699.26194	0.2
b5-H_2O	$C_{32}H_{37}O_{11}N_{6}$	681.25111	0.6
b4	$C_{28}H_{34}O_{9}N_{5}$	584.23508	0.0
b4-H_2O	$C_{28}H_{32}O_{8}N_{5}$	566.22356	1.7
y10	$C_{58}H_{68}O_{22}N_{13}$	1298.45703	2.0
y10-H_2O	$C_{58}H_{66}O_{21}N_{13}$	1280.44768	1.1
y9	$C_{54}H_{63}O_{21}N_{12}$	1215.41726	4.3
y8	$C_{43}H_{53}O_{20}N_{10}$	1029.34288	0.3
y8-H_2O	$C_{43}H_{51}O_{19}N_{10}$	1011.33148	1.2
y7	$C_{39}H_{48}O_{17}N_{9}$	914.31488	1.5
y7-H_2O	$C_{39}H_{46}O_{16}N_{9}$	896.30461	1.2
y6	$C_{35}H_{43}O_{14}N_{8}$	799.28788	1.8

CDAs and daCDAs in droplets from *Streptomyces violaceoruber* in single culture and co-culture with *Streptomyces* sp. MG7-G1

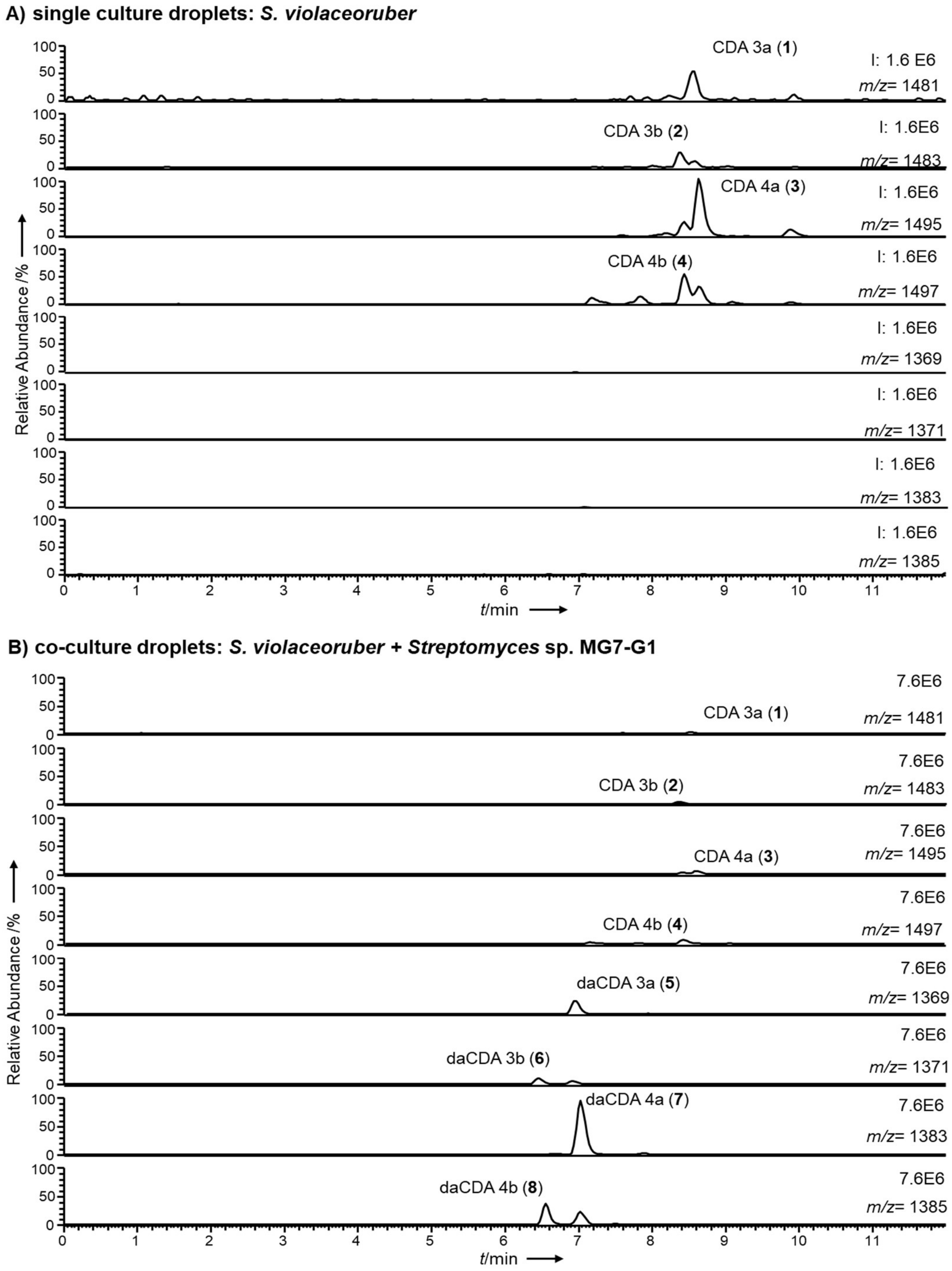

Figure S10: Comparison of ion traces of RP18 UHPL-chromatograms of exudates from A) *S. violaceoruber* grown in single-culture and B) *S. violaceoruber* grown together with *Streptomyces* sp. MG7-G1 on SFM agar plates for 18 d. All ion traces are depicted at the same intensity scale for A) 2.0 E6 and B) 9.0 E6, respectively.

Transport of compounds from the medium (fluorescein) into the droplets

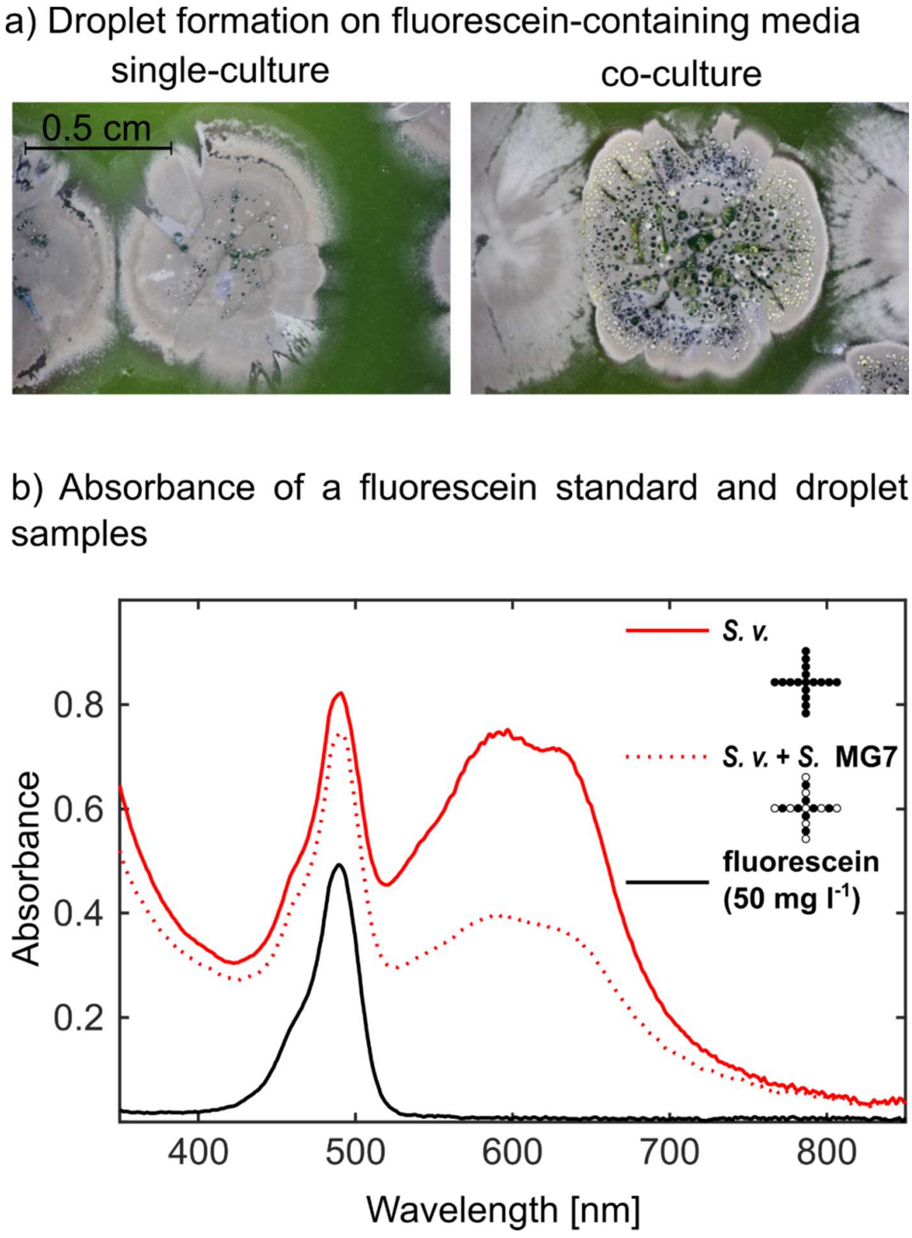

Figure S11: A) Droplet formation by *S. violaceoruber* in single-culture and co-culture with *Streptomyces* sp. MG7-G1 on fluorescein-containing SFM agar plates (50 mg l^{-1}). B) Absorbance spectra of a fluorescein standard (50 mg l^{-1}) and droplet samples of *S. violaceoruber* (*S.v.*: *S. violaceoruber, S.* MG7 *Streptomyces* sp. MG7-G1).

LC-MS ion traces for CDAs and daCDAs of *Streptomyces* sp. MG7-G1 exudate

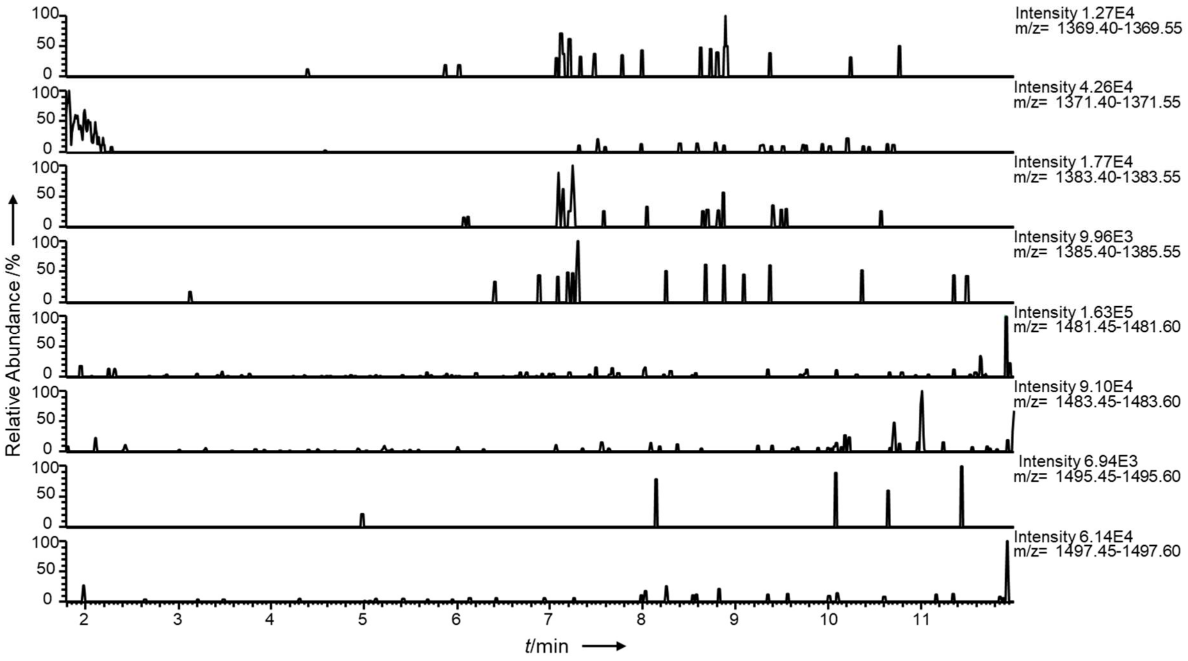

Figure S12: LC-MS ion traces for CDAs and daCDAs of *Streptomyces* sp. MG7-G1 exudate (15 d) showing no formation of CDAs or daCDAs by *Streptomyces* sp. MG7.
